# *Mark4* ablation attenuates pathological phenotypes in a mouse model of tauopathy

**DOI:** 10.1093/braincomms/fcae136

**Published:** 2024-04-17

**Authors:** Grigorii Sultanakhmetov, Sophia Jobien M Limlingan, Aoi Fukuchi, Keisuke Tsuda, Hirokazu Suzuki, Iori Kato, Taro Saito, Adam Z Weitemier, Kanae Ando

**Affiliations:** Department of Biological Sciences, Graduate School of Science, Tokyo Metropolitan University, Tokyo 192-0397, Japan; Department of Biological Sciences, Graduate School of Science, Tokyo Metropolitan University, Tokyo 192-0397, Japan; Department of Biological Sciences, Graduate School of Science, Tokyo Metropolitan University, Tokyo 192-0397, Japan; Department of Biological Sciences, Graduate School of Science, Tokyo Metropolitan University, Tokyo 192-0397, Japan; Department of Biological Sciences, Graduate School of Science, Tokyo Metropolitan University, Tokyo 192-0397, Japan; Department of Biological Sciences, Graduate School of Science, Tokyo Metropolitan University, Tokyo 192-0397, Japan; Department of Biological Sciences, Graduate School of Science, Tokyo Metropolitan University, Tokyo 192-0397, Japan; Department of Biological Sciences, School of Science, Tokyo Metropolitan University, Tokyo 192-0397, Japan; Department of Biological Sciences, Graduate School of Science, Tokyo Metropolitan University, Tokyo 192-0397, Japan; Department of Biological Sciences, School of Science, Tokyo Metropolitan University, Tokyo 192-0397, Japan; Department of Biological Sciences, Graduate School of Science, Tokyo Metropolitan University, Tokyo 192-0397, Japan; Department of Biological Sciences, School of Science, Tokyo Metropolitan University, Tokyo 192-0397, Japan

**Keywords:** *Mark4*, Alzheimer’s disease, tauopathy, neurodegeneration, astrogliosis

## Abstract

Accumulation of abnormally phosphorylated tau proteins is linked to various neurodegenerative diseases, including Alzheimer’s disease and frontotemporal dementia. Microtubule affinity-regulating kinase 4 (MARK4) has been genetically and pathologically associated with Alzheimer’s disease and reported to enhance tau phosphorylation and toxicity in *Drosophila* and mouse traumatic brain-injury models but not in mammalian tauopathy models. To investigate the role of MARK4 in tau-mediated neuropathology, we crossed P301S tauopathy model (PS19) and *Mark4* knockout mice. We performed behaviour, biochemical and histology analyses to evaluate changes in PS19 pathological phenotype with and without Mark4. Here, we demonstrated that *Mark4* deletion ameliorated the tau pathology in a mouse model of tauopathy. In particular, we found that PS19 with *Mark4* knockout showed improved mortality and memory compared with those bearing an intact *Mark4* gene. These phenotypes were accompanied by reduced neurodegeneration and astrogliosis in response to the reduction of pathological forms of tau, such as those phosphorylated at Ser356, AT8-positive tau and thioflavin S-positive tau. Our data indicate that MARK4 critically contributes to tau-mediated neuropathology, suggesting that MARK4 inhibition may serve as a therapeutic avenue for tauopathies.

## Teaser

Microtubule-affinity regulating kinase 4 (MARK4) is a tau kinase genetically linked to risks of Alzheimer’s disease. This study shows that lowering MARK4 levels reduces pathological tau aggregates and astrogliosis and ameliorates neurodegeneration and behavioural deficits in a mouse model of tauopathy, suggesting MARK4's critical role in disease pathogenesis.

## Graphical Abstract

**Graphical Abstract:**
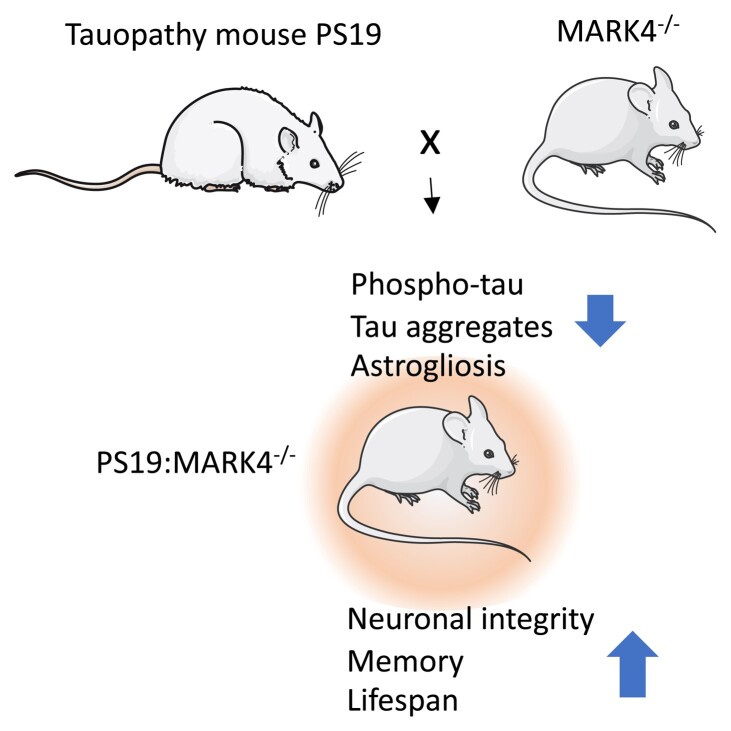

## Introduction

The abnormally phosphorylated form of the microtubule-associated protein tau has been identified as a major component of paired helical filaments in neurofibrillary tangles (NFTs) and plaque neurites in the brains of patients with Alzheimer’s disease.^1-3^ NFT depositions have been associated with cognitive decline and pathology severity in Alzheimer’s disease,^4,5^ which are the most prevalent causes of aging-associated dementia.^6^ Under physiological conditions, tau regulates microtubule stability in the axon, whereas in disease, it is hyperphosphorylated and aggregates.^7^

Among its many phosphorylation sites, those located in the tau microtubule-binding repeats, such as Ser262 and Ser356, regulate its physiological and pathological functions.^8^ High Ser262 phosphorylation has been observed from early-stage Alzheimer’s disease in pre-NFT neurons^9^ and correlates with the propagation of tau pathology.^10^ Tau phosphorylation at Ser262 and Ser356 was previously shown to promote phosphorylation at other Alzheimer’s disease-associated phospho-epitopes such as AT100 (phosphorylation at Thr212 and Ser214) and AT8 (phosphorylation at Ser202 and Thr205).^11^ Tau phosphorylation at Ser262 and Ser356 affects its interactions with chaperone complexes and degradation,^12,13^ intracellular distribution,^14^ and liquid–liquid phase separation.^15^ Substitution of these sites by non-phosphorylatable alanines dramatically reduces tau toxicity in *Drosophila* models,^11,16,17^ suggesting that phosphorylation at these sites is critical for tau toxicity.

Microtubule affinity-regulating kinase 4 (MARK4) belongs to the Par-1/MARK family, which constitutes evolutionarily conserved Ser/Thr kinases that phosphorylate microtubule-associated proteins, including tau, to regulate microtubule-dependent transport and stability.^18-21^ Previous studies reported that MARK3 and MARK4 are sequestered to granulovacuolar degeneration bodies along with tau phosphorylated at Ser262 in patients with Alzheimer’s disease,^22^ and elevation of the MARK4–tau interaction correlates with Braak stages.^23^ Moreover, genomic studies showed that a *Mark4 de novo* genetic variant was linked to early-onset Alzheimer’s disease,^24^ and Alzheimer’s disease-linked single-nucleotide polymorphisms were identified within the *Mark4* gene by a Bayesian genome-wide association study.^25^ In *Drosophila*, Par-1 overexpression enhances human tau toxicity, whereas Par-1 suppression mitigates it.^11,26^ Previously, we showed that expression of human MARK4 also enhances tau toxicity in a *Drosophila* model,^27,28^ suggesting mediation of tau abnormality by MARK4. Other studies have uncovered additional *Mark4* functions in mouse models of obesity^29^ and ischaemic brain injury.^30^ Nevertheless, the role of MARK4 in a mammalian tauopathy model has not been investigated.

Here, we examined MARK4 involvement in the disease pathogenesis of a tauopathy mouse model (PS19). By combining PS19 with a *Mark4* knockout genetic background, we demonstrated that *Mark4* deficiency significantly improved the lifespan and memory of the PS19 model. We found that although *Mark4* knockout did not affect tau phosphorylation at Ser262, it decreased Ser356 phosphorylation and reduced the abundance of AT8 phospho-epitopes and thioflavin S-positive aggregates. Interestingly, *Mark4* deletion mitigated astrogliosis in the brains of both PS19 and aged non-transgenic mice. Our results demonstrate that lowering MARK4 levels is sufficient to ameliorate the tauopathy phenotype in a mouse model, suggesting its critical involvement in neurodegenerative pathology.

## Materials and methods

The study was approved by the Research Ethics Committee of Tokyo Metropolitan University (approval numbers: A5-5, A5-6, A4-6, A4-23 and A3-11). All animal experiments were performed according to the Tokyo Metropolitan University animal experimentation guidelines and Science Council of Japan guidelines.

### Animals

*Mark4* knockout-mouse cryo-preserved spermatozoa (strain name: C57BL/6NCrl- *Mark4*^em1^(IMPC)Mbp/Mmucd, RRID: MMRRC_043405-UCD) were purchased from the Mutant Mouse Resource and Research Center at the University of California at Davis. In this strain, exons 3, 4 and the flanking splicing regions of the *Mark4* gene have been deleted using CRISPR/Cas9 gene editing. Litters were recovered by RIKEN BioResource Research Center (Tsukuba, Ibaraki, Japan). Mice expressing the human 1N4R tau protein bearing the frontotemporal dementia-associated P301S mutation [PS19; strain name: B6; C3-Tg (Prnp-MAPT*P301S) PS19Vle/J, RRID: IMSR_JAX:100010] under the prion promoter were purchased from Jackson Laboratories (Bar Harbor, Maine, USA). We used heterozygous PS19 male mice in this study.

**Figure 1:**
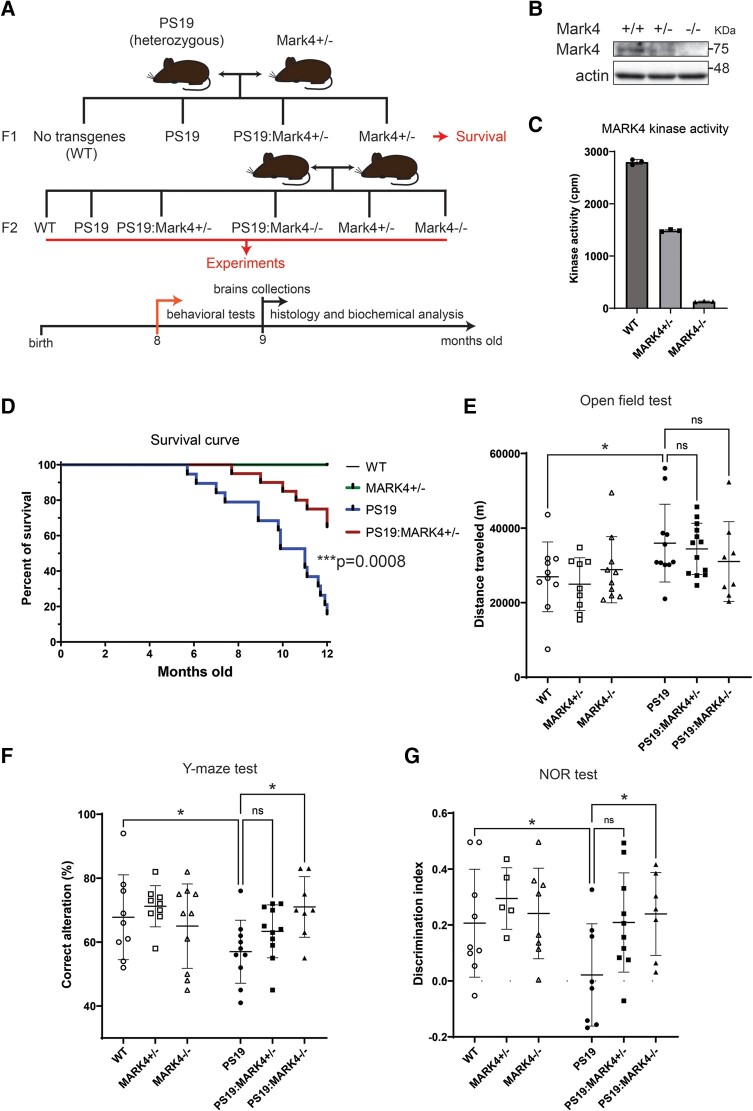
**MARK4 knockout prolonged lifespan and rescued memory deficits in PS19 mice**. (**A**) Transgenic mouse breeding scheme and experimental timeline. (**B**) Western blot of MARK4 from total brain lysates of WT (*Mark4^+/+^*), *Mark4^+/−^* and *Mark4^−/−^* mice. See Supplementary Fig. 1 for uncropped blots. (**C**) The kinase activity of MARK4 immunoprecipitated from WT, *Mark4^+/−^* and *Mark4^−/−^* mouse brain. The activity was measured as counts per minute of [g-^32^P]ATP incorporation into substrate peptides. Data represent the mean ± SD. (**D**) Kaplan–Meier survival curve of PS19 compared with PS19:*Mark4^+/−^* from the first generation (F1) of cross PS19 and *Mark4^+/−^*. The end-point for survival experiments was 12 months of age: 16% of the PS19 and 65% of PS19:*Mark4^+/−^* mice survived by this time. PS19 and PS19:*Mark4^+/−^* curves were compared using a Mantel–Cox test (***P=0.0008). N=15 (WT), N=15 (*Mark4^+/−^*), N=19 (PS19) and N=20 (PS19:*Mark4^+/−^*). (**E**) Total distance travelled within 10 min in the open field test. Data represent the mean ± SD. N=8 to N=13 mice/group. Two-way ANOVA was used to investigate the effect of the PS19 genotype (P=0.0041). *P<0.05; Tukey’s multiple comparisons test. (**F**) Percentage of correct alteration in the Y-maze spontaneous alteration test. Data represent the mean ± SD. N=8 to N=11 mice/group. Two-way ANOVA revealed the interaction effect between PS19 and *Mark4* knockout mice (P=0.0413). *P<0.05; Tukey’s multiple comparisons test. (**G**) The discrimination index was quantified to assess recognition memory in WT and PS19 mice in the presence or absence of *Mark4*. Data represent the mean ± SD. N=5 to N=10 mice/group. *P<0.05; two-way ANOVA with Tukey’s multiple comparisons test.

All mice were bred in the Tokyo Metropolitan University animal facility in a special pathogen-free area with a 12:12-h light/dark cycle and free access to food (PicoLab mouse diet 20, 5058) and water. *Mark4* knockout and PS19 mice were crossed to obtain littermates *Mark4^+/+^* [wild type (WT)], *Mark4^+/−^*, *Mark4^−/−^*, PS19, PS19:*Mark4^+/−^* and PS19:*Mark4^+/−^*. The first generation was obtained by crossing PS19 with *Mark4^+/−^*, and the second was obtained by crossing PS19:*Mark4^+/−^* with *Mark4^+/−^*. We used first-generation littermates for the survival assay and second-generation littermates for the behavioural, histological and biochemical assays (breeding scheme: Fig. 1A). Genotypes were confirmed by polymerase chain reaction (PCR) of tail DNA according to the manufacturer’s genotyping protocols using the primers listed in Table 1. Ataxia, or reaching the age of 12 months, was considered an end-point in survival experiments. Mice that developed ataxia were not used in any experiments. For histological experiments, mice were deeply anaesthetized with a double dose of 0.3-mg/kg medetomidine, 4.0-mg/kg midazolam and 5.0-mg/kg butorphanol tartare.^31^

**Table 1:** Primers

Gene	Primer name	Sequence 5′ → 3′
MARK4	Common forward	GCATGTGAACCTGTGTGAAAGGAG
MARK4	Wild-type reverse	TCAATGATCTTAATAGCGACCTGTGG
MARK4	Mutant reverse	CCCTGATACTGATCCCAGCTCCAG
P301S Tau	Common forward	TTGAAGTTGGGTTATCAATTTGG
P301S Tau	Wild-type reverse	TTCTTGGAACACAAACCATTTC
P301S Tau	Mutant reverse	AAATTCCTCAGCAACTGTGG

### Behavioural tests

PS19 mice are known to manifest cognitive impairments at the age of 6 months.^32^ Mice of different genotypes were randomly assessed on different experimental days. Experiments were performed during the light cycle in a space with dim illumination by experimenters blinded to mice genotypes on the experimental day and during analysis for all the behavioural assays. For behavioural assays, mice were habituated to the experimental space at least 30 min before assay initiation.

#### Open field assay

Mice were allowed to explore a novel white acrylic 40 × 40 × 40 cm box for 10 min. The activity was recorded on a 720p web camera. Tracking software (ToxTrack) was used to evaluate the travel distance and time spent in a 20 × 20 cm middle area of the arena. The box was wiped with 70% EtOH prior to usage by each individual mouse. We excluded mice from experiments (∼1–3 per genotype) that showed abnormal behaviour such as intensive jumping, tail rattling and/or sitting at a corner for more than half of the experimental time. The open field test was considered a habituation phase for the novel object recognition assay, which was performed the next day.

#### Novel object recognition assay

Experiments were performed according to a previously published protocol.^33^ Briefly, the day after the open field assay, mice were placed in the arena containing two identical objects. They were allowed to explore either or both objects for a total of 20 s within a maximum 10-min session. Twenty-four hours later, mice were placed in the arena containing one familiar and one novel object and again allowed to explore for 20 s within a 10-min session. Mice were considered to explore objects when their nose was headed to the object’s direction at a 2-cm distance. Then, the time spent exploring each object was analysed. The discrimination index (DI) was calculated using the following equation:


$$\mathrm{DI}=\frac{T_{\mathrm{novel}}-T_{\mathrm{old}}}{T_{\mathrm{novel}}+T_{\mathrm{old}}},$$

where *T*_novel_ and *T*_old_ are the times spent observing novel and old objects, respectively. Mice that did not show any interest in objects, i.e. they explored all objects for <20 s within a 10-min session, were excluded from the experimental analysis.

#### Y-maze assay

We observed mouse behaviour in the Y-maze to evaluate spatial working memory in our transgenic mice, as previously described.^32^ Briefly, we placed a mouse into the centre of the Y-maze and allowed it to observe the maze for 10 min. The video was recorded for every mouse to analyse their behaviour. The Y-maze has 35-cm arms with 10-cm height and 5-cm width. A successful alternation between arms is recorded when mice do not return to previously explored arms, and the percentage of successful alternation (%SA) was calculated using the following equation:


$$\text{\%}\mathrm{SA}=\frac{A_{\mathrm{successive}}}{A_{\mathrm{total}}-2},$$

where *A*_successive_ and *A*_total_ represent the amounts of successive and total alternations, respectively. We subtracted the first two alternations because they could not be successful or wrong. Mice that climbed onto walls or jumped out from the maze were excluded from the analysis.

### Histology

Mice were deeply anaesthetized with a double dose of 0.3-mg/kg medetomidine, 4.0-mg/kg midazolam and 5.0-mg/kg butorphanol tartare.^31^ Then, mice were perfused with ice-cold phosphate-buffered saline (PBS) following 4% paraformaldehyde/PBS. Mouse brains were extracted and fixed in 4% paraformaldehyde/PBS solution for 24 h. Next, brains were immersed in 30% sucrose in PBS until the brain sunk to the bottom of the 15-mL tube. Brains were sliced in 40-μm sections using a Leica cryostat CM 1510 S (Wetzlar, Germany). The sections were immersed in cryoprotectant solution (30% ethylene glycol and 20% glycerol in PBS) and kept at −20°C until further use. Mouse brain slices were observed using a Keyence BZ-X710 (Osaka, Japan) epifluorescence microscope and a Nikon AX/AX R confocal microscope (Tokyo, Japan).

#### Immunofluorescence

We performed standard free-floating mouse brain section staining to examine the effect of *Mark4* ablation on P301S tau levels, gliosis and neurodegeneration in PS19 mice.^34^ Briefly, mouse brain sections were washed in PBS, followed by permeabilization with 0.1% Triton X-100. Samples were blocked in 5% normal goat or donkey serum, depending on the host in which the secondary antibodies used were raised, for 1 h at room temperature. Then, sections were incubated with primary antibodies overnight at 4°C, followed by incubation for 2 h with secondary antibodies at room temperature and counterstaining with DAPI. Samples were stored in the dark at 4°C prior to being imaged. The primary and secondary antibodies used are listed in Table 2.

**Table 2:** Antibody list

Antibody	Distributor, reference	Host	WB dilution	IHC dilution	RRID
Anti-MAP2	Millipore, ab5622	Rabbit	na	1:500	AB_91939
Anti-PSD95	DSHB, K28/43	Mouse	na	1:500	AB_2877189
Anti-GAPDH	Novus, NB100-56875	Rabbit	1:3000	na	AB_838305
Anti-pSer262 tau	Abcam, ab131354	Rabbit	na	1:500	AB_11156689
Anti-pSer356 tau	Abcam, ab75603	Rabbit	na	1:200	AB_1310736
Anti-human tau, HT7	Thermo Fisher Scientific, MN1000	Mouse	1:5000	1:1000	AB_2314654
Anti-MARK4	Cell Signaling Technology, 4834	Rabbit	1:250	na	AB_2140610
Anti-pSer202/pThr205 tau, AT8	Thermo Fisher Scientific, MN1020	Mouse	1:1500	1:500	AB_223647
Anti-tau, Tau5	Millipore, MAB361	Mouse	1:2000	na	AB_94944
Anti-GFAP	GeneTex, GTX89226	Goat	1:2000	1:500	AB_10724708
Anti-Iba1	FUJIFILM Wako, 011-27991	Goat	na	1:500	AB_2935833
Anti-mouse IgG	Dako, P0447	Goat	1:4000	na	AB_2617137
Anti-rabbit IgG	Dako, P0399	Goat	1:4000	na	AB_2617141
Anti-goat IgG	Dako, P0449	Rabbit	1:4000	na	AB_2617143
Anti-rabbit IgG, alexa fluor 488	Thermo Fisher Scientific, A11008	Goat	na	1:500	AB_143165
Anti-mouse IgG, alexa fluor 546	Thermo Fisher Scientific, A11003	Goat	na	1:500	AB_2534071
Anti-goat IgG, alexa fluor 488	Abcam, ab150133	Donkey	na	1:500	AB_2832252

na, not applicable.

#### Thioflavin S staining

Thioflavin S staining was performed to analyse tau tangles in the brains of tauopathy model mice upon MARK4 protein ablation. Briefly, after washing, slices were mounted on a glass slide and allowed to completely dry on a heat plate at 42°C, followed by incubation with 0.5 mM thioflavin S (Merck, T1892) in 50% ethanol for 7 min.^35,36^ The number of thioflavin S-positive puncta was quantified using default thresholding segmentation and measure-particle functions in ImageJ (U. S. National Institute of Health, Bethesda, Maryland, USA).

### Biochemical analysis

At 9 months of age, mice were sacrificed by cervical dislocation, and their brains were collected, snap-frozen in liquid nitrogen and kept until further use at −80°C.

#### *In vitro* kinase assay

*In vitro* kinase assay was performed to validate kinase activity levels in our *Mark4* knockout mice. Mouse whole brains were homogenized in 10 vol (1:10 weight/volume ratio) of 3-morpholino-propane-sulfonic acid (MOPS) buffer (20 mM MOPS, pH 6.8, 1 mM EGTA, 0.1 mM EDTA, 0.3 M NaCl, 1 mM MgCl_2_, 0.5% Nonidet P-40 and 1 mM DTT + proteinase inhibitors: 0.2 mM Pefabloc SC and 1-mg/mL leupeptin and phosphatase inhibitors: 10 mM β-glycerophosphate and 5 mM NaF) with a Teflon pestle homogenizer and centrifuged at 10 000 × *g* for 15 min at 4°C to collect the extract as a supernatant. MARK4 was immunoprecipitated from brain lysates with an anti-MARK4 antibody (Novus Biologicals, NB100-1013) and protein-G Dynabeads (Thermo Fisher Scientific). Its kinase activity was measured using Chktide (SignalChem) and [g-^32^P]ATP as substrates. The incorporation of ^32^P into Chktide was quantified using a liquid scintillation counter (Beckman Coulter).

#### Western blotting

Western blots were performed to analyse the relative protein levels of total and fractionated tau. One of the two brain hemispheres was homogenized in 10 vol of radioimmunoprecipitation assay (RIPA) buffer (50 mM Tris base, pH 8.0, 0.15 M NaCl, 1% Nonidet P-40, 0.5% Na-deoxycholate, 5 mM EDTA, 1 mM EGTA and 0.1% SDS + proteinase inhibitors and phosphatase inhibitors) using a Teflon pestle homogenizer and centrifuged at 15 800 × *g* for 20 min at 4°C to collect the extract as a supernatant. Protein concentration was measured by Bradford protein assay (Pierce, 23200) and adjusted to 2 µg/μL. Then, protein homogenates were mixed with 2× loading buffer (124.8 mM Tris base, pH 6.8, 4% SDS, 20% glycerol, 0.2-mg/mL bromophenol blue, 10% 2-mercaptoethanol + proteinase inhibitors and phosphatase inhibitors) to a final concentration of 1 µg/μL, and 10 μg of protein from each sample was loaded to a 10% gel for SDS–PAGE. After gel electrophoresis, proteins were transferred to PVDF membranes, which were blocked in 5% bovine serum albumin/TBST (0.1 M Tris base pH 7.6, 0.15 M NaCl, 0.05% Tween 20) and then incubated with the designated primary and secondary antibodies described in Table 2. The band signals were visualized using Immobilon Western Chemiluminescent HRP Substrate (Millipore) in Fusion SL (Vilber, France).

#### Tau protein extraction

Sequential extraction of tau protein was performed to test the effect of MARK4 on tau protein solubility. We performed fractionation analysis as previously described for PS19 mice.^34,37^ Briefly, one brain hemisphere was homogenized in 5 vol high-salt reassembly buffer (HS-RAB: 100 mM MES, pH 7.0, 1 mM EGTA, 0.5 mM MgSO_4_, 0.75 M NaCl and 0.1 mM EDTA + proteinase inhibitors and phosphatase inhibitors) using a Teflon pestle homogenizer, and the homogenate was centrifuged at 50 000 × *g* in an Optima MAX-TL ultracentrifuge (Beckman Coulter, Brea, CA, USA) for 40 min at 4°C to collect the supernatant as an HS-RAB soluble fraction. The pellet was homogenized in 1 M sucrose/RAB buffer, and the solution was centrifuged at 50 000 × *g* for 20 min, followed by pellet homogenization in 1 vol RIPA buffer and centrifugation at 50 000 × *g* for 20 min at 4°C to collect the supernatant as a RIPA soluble fraction. Next, we extracted a RIPA-insoluble pellet with 1 vol of cold 70% formic acid solution and centrifuged it at 15 800 × *g* for 20 min at 4°C to collect the supernatant as an FA soluble fraction. The FA fraction was diluted in 1:10 (*v*/*v*) neutralization buffer (1 M Tris base and 0.5 M Na_2_HPO_4_), and the pH was checked using pH strips (Merck). All fractions were processed for western blotting as described above.

### Analysis

Experimenters were blind regarding mice genotype during data collection and manual analysis. The study design was made to create a random distribution regarding genotype for data and sample collection. Briefly, we crossed PS19:*Mark4*^+/−^ and *Mark4*^+/−^ to get littermates with different genotypes, which we split two to four per cage in random order; therefore, when mice reached the desired age for experiments, they were assayed or collected in random order regarding genotype. For the open field test, the travelled distance and time spent in the middle area (20 × 20 cm) were quantified using ToxTrack version 2.96.^38,39^ Western blot and microscopy data were analysed using ImageJ version 1.53c.^40,41^ Band intensities were quantified using gel-selection and plot-line functions followed by band peak underline area measurements. In immunohistochemistry experiments, the area covered by astroglia and microglia was quantified using default thresholding segmentation and measured-particle functions. For other signals, integrated intensities were evaluated using the measurement function, followed by normalization to the region of interest size. Data are represented as fold changes, normalized to average levels of control (PS19 or WT groups).

### Statistical analysis

Data analysis was performed in GraphPad Prism 9 and GPower 3.1. We computed the required total sample size of 54 for behavioural experiments (medium effect size f=0.5,^42^ desired power 1−β=0.9, α=0.05), which means nine mice per experimental group on average. Our ‘dummy’ test for histological analysis predicted five mice per genotype for 0.9 power—the actual power was between 0.8 and 0.9 for experiments that showed differences among means. The Mantel–Cox test was used to compare survival curves between PS19 and PS19:*Mark4^+/−^* animals. A two-way analysis of variance (ANOVA) test was used for the behavioural assay, where *Mark4* deficiency was analysed in WT and PS19 mice followed by a Tukey’s multiple comparisons test of the means between each group. For other tests, in which the effect of *Mark4* deficiency was analysed in PS19 mice, and WT was used as a negative control, one-way ANOVA was implemented, followed by Holm–Sidak’s or Dunnett’s multiple comparison tests among the means or between the means of the PS19 and the PS19:*Mark4^+/−^* or PS19:*Mark4^−/−^* groups, respectively. Data normality and homogeneity were tested by D'Agostino–Pearson omnibus (K2) and Barlett’s (one-way ANOVA) or Spearman’s (two-way ANOVA) tests, correspondingly. For thioflavin S staining analysis, we performed a Kruskal–Wallis test followed by a Dunn’s multiple comparisons test. Statistical tests for each experiment are specified in the figure legends. Differences were considered statistically significant when P<0.05.

## Results

### *Mark4* knockout prolonged lifespan and rescued memory deficits in PS19 mice

To test whether MARK4 suppression affects the abnormal behavioural phenotype and mortality of PS19 mice, we crossed them with *Mark4* knockout mice (Fig. 1A, two generations F1 and F2). Homozygous *Mark4* knockout mice (hereafter referred to as *Mark4^−/−^*) are fertile and develop to adulthood without apparent abnormalities.^43,44^ In addition to tail PCR genotyping, we confirmed the absence of MARK4 in *Mark4^−/−^* mice by western blotting and *in vitro* kinase assays (Fig. 1B and C; Supplementary Fig. S1). PS19 mice have a shorter lifespan,^34^ and in our colony, they had a median lifespan of 10.9 months, while all WT and *Mark4^+/−^* mice survived until 12 months of age. We found that in the first generation from crossing PS19 and *Mark4^+/−^* mice (F1; Fig. 1A), PS19:*Mark4^+/−^* mice lived significantly longer than their PS19 counterparts (P<0.001; Fig. 1D): at 12 months, the survival rates of PS19 and PS19:*Mark4^+/−^* were 16% versus 65%, respectively. PS19:*Mark4^+/−^* and PS19:*Mark4^−/−^* obtained by crossing PS19:*Mark4^+/−^* and *Mark4^+/−^* (F2; Fig. 1A) showed less number of mice developing ataxia compared with PS19 by 9 months old (Supplementary Table S1).

Hyperactivity of PS19 mice, such as enhanced locomotion and more frequent alternations in the Y-maze,^32^ as well as enhanced locomotor activity in the open field test,^32,45^ has been previously reported. In agreement with previous studies, 8-month-old PS19 mice showed enhanced locomotor activity compared with WT mice in the open field test. PS19:*Mark4^−/−^* mice showed a shorter, albeit not significantly, travelled distance than PS19 mice (P=0.46; Fig. 1E) and spent a similar amount of time in the middle area of the arena as PS19 mice (Supplementary Fig. 2A and B).

**Figure 2:**
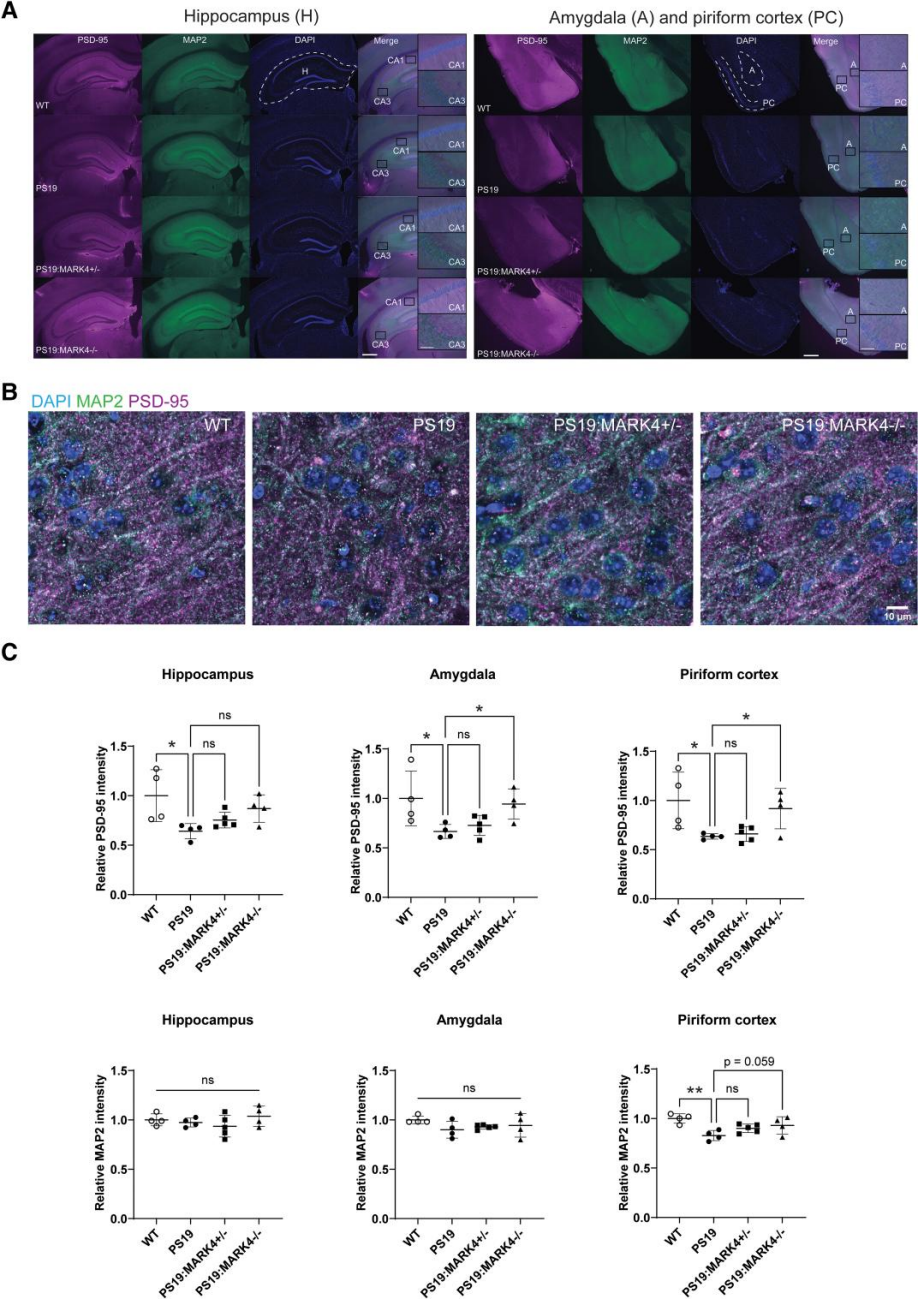
***Mark4* knockout ameliorated the loss of synapses and dendrites in PS19 mice.** (**A**) Representative images of double immunostaining of the hippocampus, amygdala and piriform cortex regions for the postsynaptic marker PSD-95 and dendritic marker MAP2. The dashed line highlights the hippocampus (H), piriform cortex (PC) and amygdala (A). Insets represent magnified CA1, CA3, amygdala (A) and piriform cortex (PC) regions. Scale bars: 500 and 50 μm (insets). (**B**) Representative laser scanning confocal microscopy images of neurons in the piriform cortex for PSD-95, MAP2 and DAPI. Separate channel images are in Supplementary Fig. 3. Scale bar: 10 μm. (**C**) Quantification of PSD-95 and MAP2 integrated intensity in the hippocampus, amygdala and piriform cortex normalized to the region area size. Fold changes are represented relative to WT levels. Data represent the mean ± SD. N=4 to N=5 mice/group. *P<0.05; one-way ANOVA with Holm–Sidak’s multiple comparisons test. Mice were 9 months old.

To assess the memory performance of our transgenic mice, we implemented the Y-maze spontaneous alteration test and novel object recognition test. The Y-maze spontaneous alteration test is designed to evaluate spatial working memory in mice.^46^ As previously reported,^32,45^ PS19 mice showed a lower number of correct alternations. Interestingly, *Mark4* copy number negatively correlated with increased performance of PS19 mice in the Y-maze spontaneous alteration test (57%, 63% and 71% of correct alternations in PS19, PS19:*Mark4^+/−^* and PS19:*Mark4^−/−^* mice, respectively), with a significant difference between PS19 and PS19:*Mark4^−/−^* mice (P<0.05; Fig. 2F). However, the number of total alternations in the Y-maze was not significantly different between experimental groups (Supplementary Fig. 2C). To assay recognition memory in our transgenic mice,^33^ we performed the novel object recognition test and observed that PS19 mice performed more poorly than WT in agreement with previous studies^32,45^ (Fig. 1G). PS19:*Mark4^+/−^* or PS19:*Mark4^−/−^* performed better than PS19: the novel object recognition memory performance scores of PS19:*Mark4^+/−^* and PS19:*Mark4^−/−^* were higher than those of PS19 (P=0.063 and P=0.044, respectively) and similar to those of WT (P=0.99 and P=0.99, respectively; Fig. 1G).

In addition, we did not observe abnormalities in the survival, open field activity or memory functions of our *Mark4* knockout mice (Fig. 1D–G; WT, *Mark4^+/−^* and *Mark4^−/−^* groups). Our findings corroborate previous studies, which did not identify pathological abnormalities in *Mark4*-deficient mice.^29,43,44^

### *Mark4* knockout ameliorated the loss of synapses and dendrites in PS19 mice

We were then interested in whether *Mark4* knockout affects neurodegeneration in PS19 mice. To this end, we analysed neurons and glia in regions involved in memory functions in the hippocampus, amygdala and piriform cortex, where we confirmed a relatively high level of human P301S tau protein in the brain of a 9-month-old PS19 mouse (Supplementary Fig. 3) as was also shown in previous studies.^34,36,47^ First, we performed immunostaining using an antibody against the postsynaptic marker PSD-95 to identify synapse density. We observed that synapse density was lower in all tested brain regions of PS19 mice (P<0.05; Fig. 2A–C and Supplementary Fig. 4; compare WT and PS19). Although PS19:*Mark4^+/−^* brains exhibited only moderate changes in PSD-95 immunoreactivity compared with PS19 animals (P>0.1; Fig. 2A–C), PS19:*Mark4^−/−^* brains displayed significantly higher PSD-95 signal in the amygdala and piriform cortex than PS19 brains (P<0.05), and the signals were as high as those in WT (P=0.97 and P=0.85, respectively; Fig. 2A–C). The average PSD-95 staining intensity in PS19:*Mark4^−/−^* mice was also higher in the hippocampus than in PS19 mice (P=0.065).

Immunostaining with MAP2 antibody was carried out to detect dendritic loss.^48^ Among the tested regions, MAP2 immunoreactivity was similar in the hippocampus and amygdala between all experimental groups (P>0.5; Fig. 2A and C). MAP2 significantly reduced only in the piriform cortex of PS19 mice compared with the WT (P<0.01), whereas it was recovered in PS19:*Mark4^−/−^* animals compared with PS19 (P=0.056; Fig. 2A–C and Supplementary Fig. 4).

### *Mark4* knockout reduced Ser356 phosphorylation levels in PS19 mice

**Figure 3:**
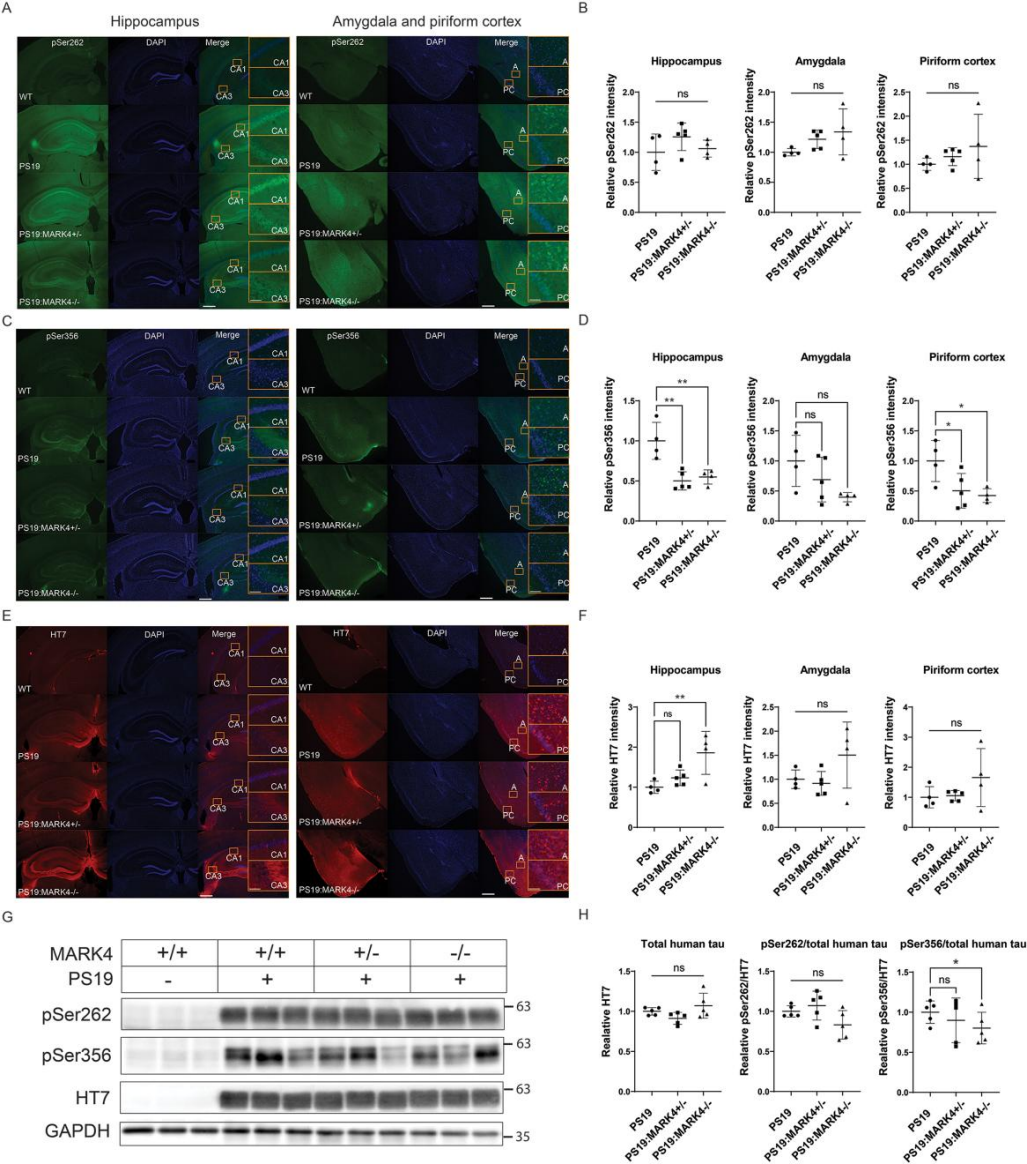
***Mark4* knockout decreased tau phosphorylation at Ser356.** (**A**) Representative images of pSer262 tau immunostaining in the hippocampus, amygdala and piriform cortex. (**B**) Graphs show the integrated intensity quantification normalized to the region area size. Fold changes are represented relative to levels in PS19 mice. Data represent the mean ± SD. N=4 to N=5 mice/group. ns, non-significant; one-way ANOVA with Dunnett’s multiple comparisons test. Insets represent the CA1, CA3, amygdala (A) and piriform cortex (PC) regions. Scale bars: 500 and 50 μm (insets). (**C**) Representative images of pSer356 tau immunostaining with (**D**) its integrated intensity quantification in the hippocampus, amygdala and piriform cortex. Data represent the mean ± SD. N=4 to N=5 mice/group. ns, non-significant; *P<0.05; **P<0.01; one-way ANOVA with Dunnett’s multiple comparisons test. Insets represent the CA1, CA3, amygdala (A) and piriform cortex (PC) regions. Scale bars: 500 and 50 μm (insets). (**E**) Representative images of total human tau (HT7) protein immunostaining with (**F**) its integrated intensity quantification in the hippocampus, amygdala and piriform cortex. Data represent the mean ± SD. N=4 to N=5 mice/group. ns, non-significant; *P<0.05; one-way ANOVA with Dunnett’s multiple comparisons test. Insets represent the CA1, CA3, amygdala (A) and piriform cortex (PC) regions. Scale bars: 500 and 50 μm (insets). (**G**) Immunoblot of human total (HT7), pSer262 and pSer356 tau from brain lysates of WT, PS19, PS19:MARK4^+/−^ and PS19:MARK4^−/−^ with (**H**) band integrated intensity quantification and normalization to GAPDH (HT7) or total tau levels (pSer262 and pSer356). Fold changes are represented relative to levels in PS19 samples. Data represent the mean ± SD. N=5 mice/group. ns, non-significant; *P<0.05; one-way ANOVA with Dunnett’s multiple comparisons test. See Supplementary Fig. 5 for uncropped blots.

MARK4 phosphorylates the KXGS motifs of the tau microtubule-binding repeats, such as at Ser262 and Ser356.^18^ Therefore, we sought to identify whether *Mark4* ablation affects tau phosphorylation at Ser262 and Ser356 (pSer262 and pSer356) in PS19 mice. Immunostaining with a tau anti-pSer262 antibody showed no differences among PS19, PS19:*Mark4^+/−^* and PS19:*Mark4^−/−^* genotypes in all tested regions (P>0.1; Fig. 3A and B). pSer356 immunolabeling was weak in CA1 and prevalent in CA3 and the dentate gyrus in the hippocampus of PS19 mice (Fig. 3C, insets). By contrast to pSer262, pSer356 signal intensity was reduced about 2-fold in PS19:*Mark4^+/−^* and PS19:*Mark4^−/−^* compared with PS19 in all tested regions (Fig. 3C and D). The reduction in pSer356 was significant for hippocampus and piriform cortex regions in PS19:*Mark4^−/−^* and PS19:*Mark4^+/−^* compared with PS19 (P<0.05; Fig. 3D).

Additionally, we analysed human tau expression with the human tau-specific antibody HT7. Immunostaining with HT7 exhibited significantly higher signal intensity in the hippocampus of PS19:*Mark4^−/−^* mice than in PS19 animals (P<0.05), whereas it displayed similar intensity in the amygdala and piriform cortex (P>0.1; Fig. 3E and F). PS19:*Mark4^+/−^* mice showed similar levels of HT7 tau as PS19 mice (P>0.1; Fig. 3E and F).

Western blotting experiments confirmed the reduction of pSer356 in PS19:*Mark4^−/−^* compared with PS19 (P<0.05), while pSer262 levels were not significantly affected (P>0.1; Fig. 3G and H and Supplementary Fig. S5; pSer356 and pSer262). Total tau levels were not significantly affected by MARK4 knockout in PS19 mice (P>0.1; Fig. 3G and H and Supplementary Fig. 5; HT7).

### *Mark4* knockout reduced AT8-positive tau levels in PS19 mice

**Figure 4:**
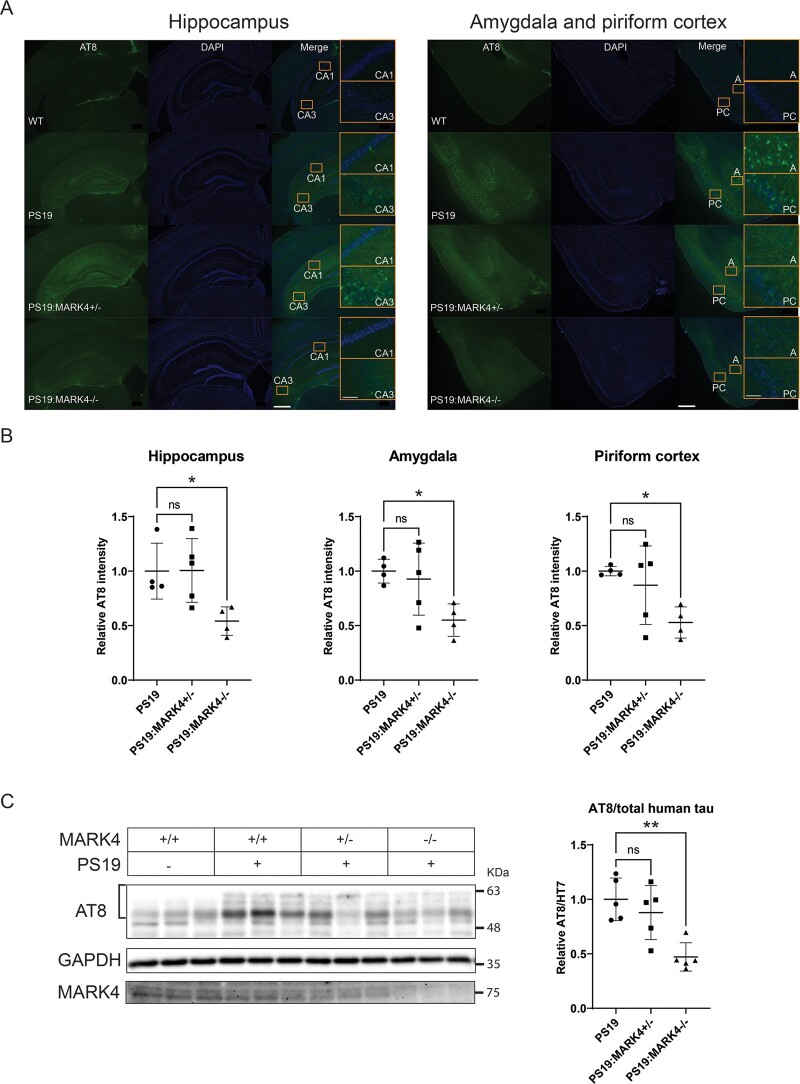
***Mark4* knockout reduced tau phosphorylation at AT8 sites in PS19 mice. (A)** Representative images of AT8 (pSer202/pThr205) immunostaining, with (**B**) integrated intensity quantification in the hippocampus, amygdala and piriform cortex. The average background signal from WT was subtracted. Fold changes are represented relative to levels in PS19. Data represent the mean ± SD. N=4 to N=5 mice/group. ns, non-significant; *P<0.05; **P<0.01; ***P<0.001; one-way ANOVA with Dunnett’s multiple comparisons test. Insets represent the CA1, CA3, amygdala (A) and piriform cortex (PC) regions. Scale bars: 500 and 50 μm (insets). (**C**) Immunoblot of AT8 and MARK4 from brain lysates of WT, PS19, PS19:MARK4^+/−^ and PS19:MARK4^−/−^ with AT8 band integrated intensity quantification (brackets) and normalization to total human tau protein levels. The average background signal from WT mice was subtracted. Fold changes are represented relative to levels in PS19 samples. Data represent the mean ± SD. N=5 mice/group. ns, non-significant; *P<0.05; one-way ANOVA with Dunnett’s multiple comparisons test. See Supplementary Fig. 6 for uncropped blots.

Immunostaining intensity using an AT8 antibody (pSer202 and pThr205) correlates with disease severity in tauopathy.^9^ PS19 mice start to exhibit an AT8 signal above baseline around 5 months of age in the hippocampus.^49^ We conducted immunohistochemistry and western blotting to evaluate the effect of *Mark4* knockout on the abundance of the AT8 phospho-epitope in PS19 mice. We observed lower AT8 staining intensity in PS19:*Mark4^−/−^* than in PS19 mice when heterozygous Mark4 knockout has no significant effect on AT8 levels (Fig. 4A–C). The reduction in AT8 was significant for all tested regions in PS19:*Mark4^−/−^* (P<0.05). Next, we compared the AT8-positive total tau levels in brain lysates. In line with immunohistochemistry experiments, a 2-fold reduction in AT8 signal in PS19:*Mark4^−/−^* mice was observed by western blotting (P<0.01; Fig. 4C and Supplementary Fig. 6). AT8-positive tau levels appeared to be, on average, reduced in PS19:*Mark4^+/−^* compared with PS19 mice; however, this difference was not significant (P>0.1; Fig. 4C). Additionally, we confirmed the presence of *Mark4* knockout in our tested groups (Fig. 4C).

### *Mark4* knockout reduced thioflavin S-positive tau aggregates in PS19 mice

**Figure 5:**
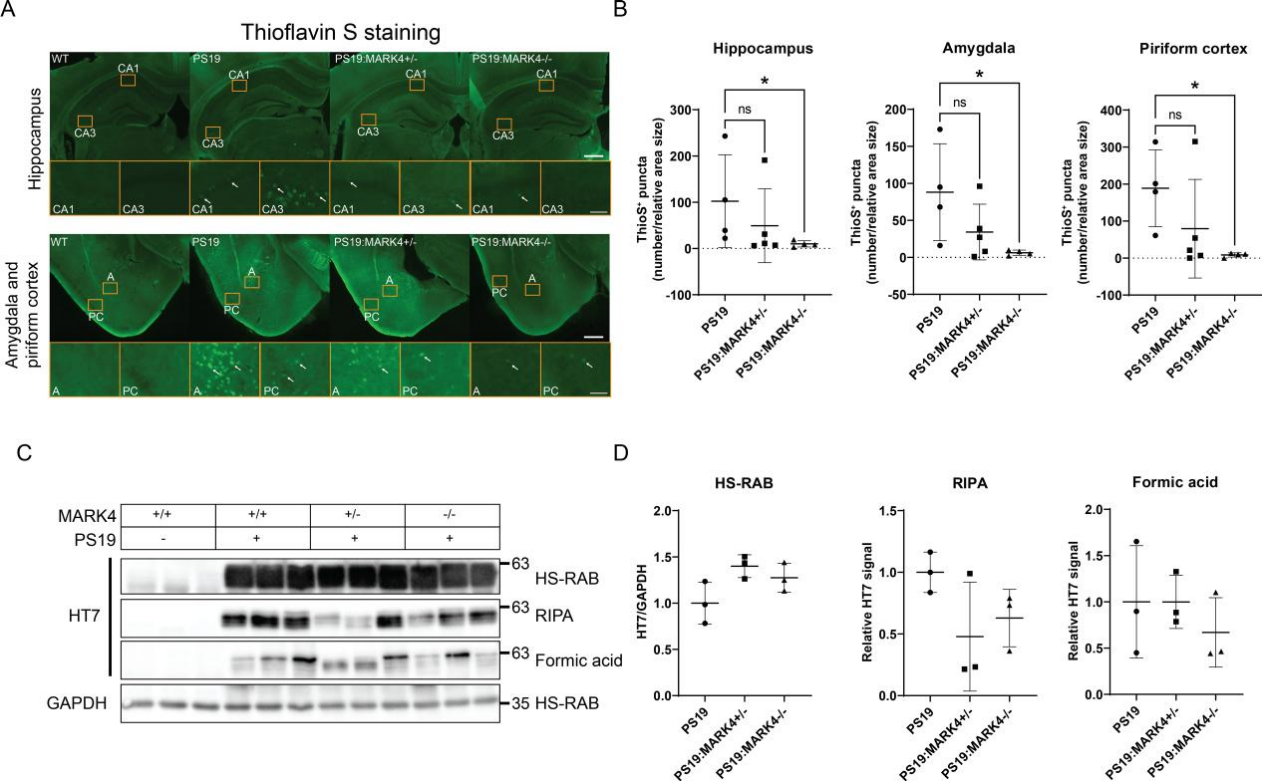
**Reduction of thioflavin S-positive tau aggregates and increasing tau solubility as a result of *Mark4* knockout in PS19 mice.** (**A**) Representative images of thioflavin S staining of the hippocampus, amygdala and piriform cortex of the mouse brain. Examples of thioflavin S-positive (ThioS^+^) puncta are labelled with arrows in the magnified panels. Panels represent CA1, CA3, amygdala (**A**) and piriform cortex (PC) regions. Scale bars: 500 and 50 μm (panels). (**B**) Quantification of the amount of ThioS^+^ puncta in the hippocampus, amygdala and piriform cortex. Data represent the mean ± SD. N=4 to N=5 mice/group. ns, non-significant; *P<0.05; Kruskal–Wallis test with Dunn’s multiple comparison test. (**C**) Western blot of total human tau (HT7) from HS-RAB, RIPA buffer, and formic acid extraction of brain lysates of WT, PS19, PS19:*Mark4^+/−^* and PS19:*Mark4^−/−^* mice with (**D**) band integrated intensity quantification normalized to GAPDH levels for HS-RAB lysate. Fold changes are represented relative to levels in PS19 samples. Data represent the mean ± SD. N=3 mice/group. No significant differences between groups were found (P>0.05). One-way ANOVA with Dunnett’s multiple comparisons test. See Supplementary Fig. 7 for uncropped blots.

Thioflavin S-positive tau depositions have been observed in the neocortex, hippocampus and amygdala at 6 months of age in PS19 mice.^34^ Thus, we sought to evaluate the effect of MARK4 suppression on NFT pathology in PS19 mice by performing thioflavin S staining. Thioflavin S staining of tau appeared as puncta (Fig. 5A, white arrows). Thioflavin S puncta were significantly fewer in PS19:*Mark4^−/−^* than in PS19 mice in all brain regions tested (P<0.05; Fig. 5B). A reduction in Thioflavin S-positive puncta was observed in PS19:*Mark4^+/−^* animals, but the difference was not significant due to variations among individuals (P>0.1). In particular, PS19:*Mark4^+/−^* mice showed almost 2-fold reduction compared with PS19, and PS19:*Mark4^−/−^* showed further reduction: whereas PS19 had more than a hundred puncta, PS19:*Mark4^−/−^* animals displayed only a dozen of puncta in average (Fig. 5B).

Next, we analysed the effect of *Mark4* knockout on tau solubility by conducting sequential protein extraction from brain lysates using buffers with different extraction strengths (HS-RAB → RIPA → formic acid; Fig. 5C), followed by western blotting with the anti-human tau antibody HT7.^34,37^ Tau in brain homogenates of PS19:*Mark4^+/−^* and PS19:*Mark4^−/−^* mice tended to be fractionated more in HS-RAB and less in RIPA or formic acid fractions compared with PS19. Tau proteins in the HS-RAB fraction from PS19:*Mark4^+/−^* and PS19:*Mark4^−/−^* mice were 40% and 28% more, respectively, whereas those in the RIPA fraction were 52% and 37% less, than in PS19 samples, respectively (Fig. 5C and D; Supplementary Fig. 7). Tau proteins from PS19:*Mark4^−/−^* samples fractionated to the formic acid fraction and were 33% 4 those in PS19 brain lysates (Fig. 5C and D). However, the difference was insignificant due to the large variation among individuals (P>0.1).

### *Mark4* knockout reduced astrogliosis in WT and PS19 mice

**Figure 6:**
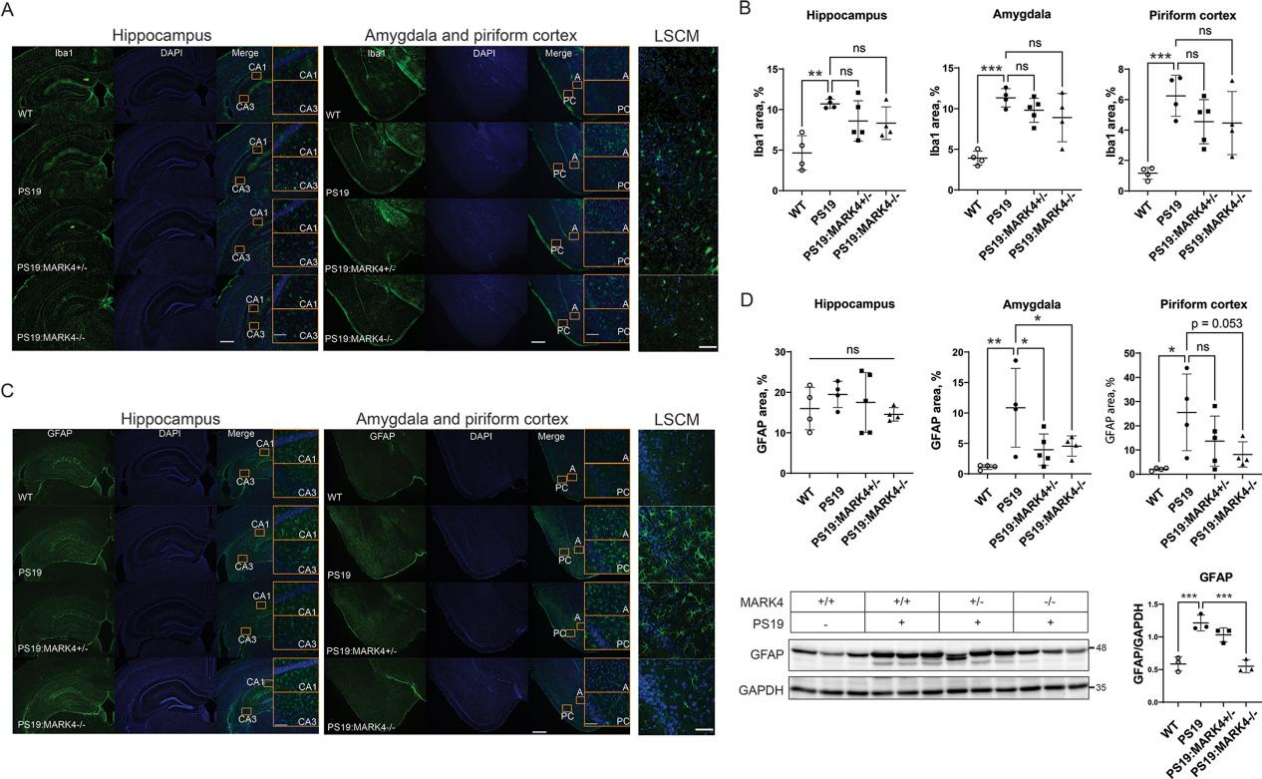
***Mark4* knockout reduced astrogliosis in PS19 mice.** (**A**) Representative images of Iba1 immunostaining with (**B**) quantification of the percentage of Iba1-positive area in the hippocampus, amygdala and piriform cortex. Data represent the mean ± SD. N=4 to N=5 mice/group. ns, non-significant (P>0.05); **P<0.01; ***P<0.001; one-way ANOVA with Holm–Sidak’s multiple comparisons test. Insets represent the CA1, CA3, amygdala (A) and piriform cortex (PC) regions. Scale bars: 500 and 50 μm (insets). LSCM, laser scanning confocal microscopy images of piriform cortex. Scale bar: 50 μm. (**C**) Representative images of GFAP immunostaining with (**D**) quantification of the percentage of GFAP-positive area in the hippocampus, amygdala and piriform cortex. Data represent the mean ± SD. N=4 to N=5 mice/group. ns, not significant (P>0.05); *P<0.05; **P<0.01; one-way ANOVA with Holm–Sidak’s multiple comparisons test. Insets represent the CA1, CA3, amygdala (A) and piriform cortex (PC) regions. Scale bars: 500 and 50 μm (insets). LSCM, laser scanning confocal microscopy images of piriform cortex region. Scale bar: 50 μm. (**E**) Immunoblot of GFAP from total brain lysate of WT, PS19, PS19:*Mark4^+/−^* and PS19:*Mark4^−/−^* mice with GFAP bands integral intensity quantification normalized by GAPDH. Data are mean ± SD. N=3 mice/group. Ns, non-significant, ***P<0.001; one-way ANOVA with Holm–Sidak’s multiple comparisons test. See Supplementary Fig. 8 for uncropped blots.

We also sought to analyse the effect of *Mark4* ablation on the activation of microglia and astroglia, which are signs of neuroinflammation. PS19 mice show neuroinflammation detected by gliosis at 6 months of age.^34^ Immunostaining with the microglial marker Iba1 revealed that although the number of microglia was increased in PS19 compared with WT mice, it was not significantly reduced upon *Mark4* ablation (P>0.1 among PS19, PS19:*Mark4^+/−^* and PS19:*Mark4^−/−^* mice; Fig. 6A and B). Glial fibrillary acidic protein (GFAP), a marker of reactive astroglia, was increased in the PS19 amygdala and piriform cortex, indicating astrogliosis. In contrast to microgliosis, *Mark4* knockout displayed a prominent effect on astrogliosis, as GFAP signals were lower in PS19:*Mark4^+/−^* and PS19:*Mark4^−/−^* than those in PS19 mice (Fig. 6C and D). We observed astrogliosis reduction in both the PS19:*Mark4^+/−^* and PS19:*Mark4^−/−^* amygdala compared with PS19 mice (P<0.05). The piriform cortex in PS19:*Mark4^−/−^* also showed a reduction in astrogliosis (P=0.053). In the hippocampus, GFAP staining was similar among WT, PS19, PS19:*Mark4^+/−^* and PS19:*Mark4^−/−^* mice (P>0.1; Fig. 6B and D). We also analysed the levels of GFAP in brain extracts by western blotting. GFAP levels were significantly reduced in PS19:*Mark4^−/−^* compared with PS19 mice (P<0.001; Fig. 6E and Supplementary Fig. 8).

**Figure 7:**
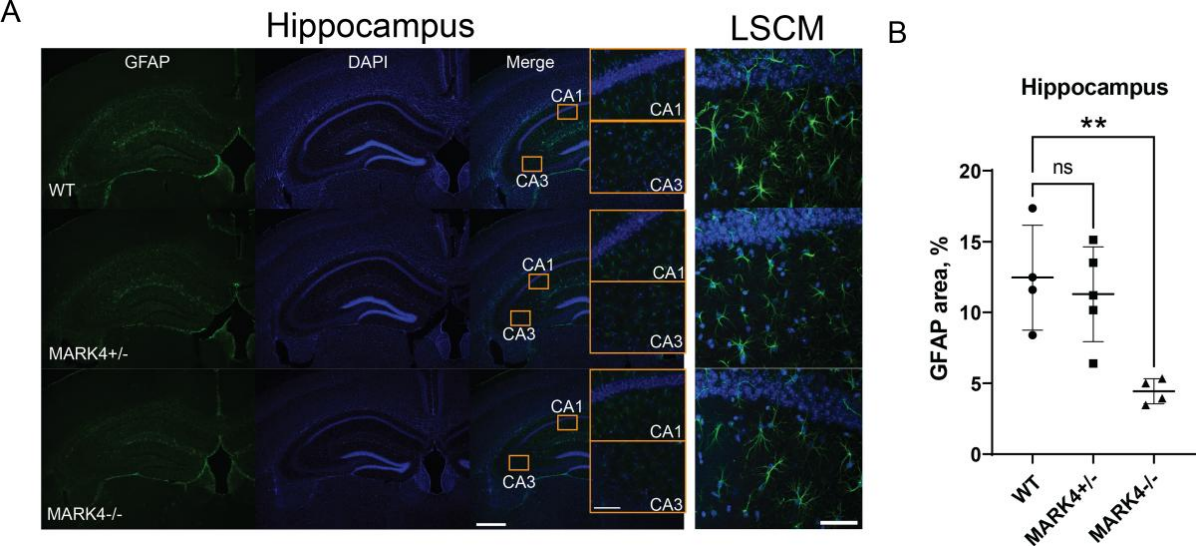
***Mark4* knockout reduced astrogliosis in the hippocampus of WT mice.** (**A**) Representative images of GFAP immunostaining with (**B**) quantification of the percentage of GFAP-covered area in the hippocampus of WT, *Mark4^+/−^* and *Mark4^−/−^* mice. Data represent the mean ± SD. N=4 to N=5 mice/group. ns, non-significant; **P<0.01; one-way ANOVA with Dunnett’s multiple comparisons test. Insets represent the CA1, CA3, amygdala (A) and piriform cortex (PC) regions. Scale bars: 500 and 50 μm (insets). LSCM, laser scanning confocal microscopy images of hippocampus CA1 region. Scale bar: 50 μm.

Since MARK4 is directly involved in inflammation processes,^50^ we were motivated to test whether *Mark4* ablation suppresses astrogliosis without human tau expression. We compared GFAP staining of reactive astroglia in the hippocampus of 9-month-old WT, *Mark4^+/−^* and *Mark4^−/−^* mice (Fig. 7A), where GFAP-positive astroglia was more abundant than in other tested regions of WT mice (Fig. 6C and D). *Mark4^+/−^* mice showed the same reactive astroglia levels as their WT counterparts (P>0.5; Fig. 7B). The GFAP signal was reduced to <50% in *Mark4^−/−^* mice than in WT mice (P<0.01; Fig. 7B), indicating that MARK4 functions independently of tau toxicity in the activation of astroglia.

## Discussion

In this study, we analysed the role of MARK4 in tau-induced neurodegeneration by crossing a tauopathy model (PS19)^32,34,45,51^ with *Mark4* knockout mice. We showed that *Mark4* deletion could decrease mortality, ameliorate memory deficits and reduce synapse and dendritic loss in PS19 mice (Figs 1 and 2). *Mark4* deficiency decreased the levels of tau phosphorylation at Ser356 and AT8 and Thioflavin S-positive aggregates (Figs 3–5). Tau solubility was also analysed biochemically with sequential protein extraction (Fig. 5C). Although *Mark4* deficiency tends to reduce tau insolubility, the difference was not statistically significant. The number of animals was limited, and analyses with more animals in the future may help to conclude the effect of *Mark4* knockout on tau solubility. We also demonstrated that *Mark4* knockout suppressed astrogliosis in the PS19 model (Fig. 6) and in the hippocampus of mice that did not express human tau protein (Fig. 7), thus revealing a novel role of MARK4 in astrogliosis. In all experiments, MARK4^−/−^ background improved PS19 phenotypes more prominently than MARK^+/−^ background, indicating dosage-dependent effects (Figs 1–7).

Previous reports suggest that a reduction in tau phosphorylation correlates with a lower accumulation of pathological aggregates.^36,45,51-53^ As a Par-1/MARK protein family member, MARK4 phosphorylates tau at the serine residues of KXGS motifs of microtubule-binding repeats, including Ser262 and Ser356.^20^ Tau phosphorylation at Ser262 was detected in pre-NFT neurons when prominent staining was observed with the 12E8 antibody, which recognizes both pSer262 and pSer356 tau.^9^ This finding suggests that tau phosphorylation at these sites promotes tau aggregation. However, tau phosphorylation at Ser262 has been reported to prevent tau aggregation,^54^ and phosphorylation sites that drive tau aggregation do not include pSer262.^55^ Interestingly, the effect of Ser356 phosphorylation on tau aggregation has only been investigated in the presence of pSer262. Here, we found that *Mark4* knockout decreased the pSer356 without affecting pSer262 levels in PS19 animals (Fig. 3). pSer356 reduction was correlated with less AT8-positive tau and fewer thioflavin S-positive tau aggregates (Figs 4 and 5). These results suggest that reduced tau phosphorylation at Ser356 may block its aggregation. They are also in agreement with a previous study that demonstrated that the NUAK Family Kinase 1 exclusively phosphorylated Ser356, and its ablation reduced NFT formation and rescued memory deficits and synaptic plasticity in PS19 mice.^51^ Interestingly, both MARK4 and NUAK Family Kinase 1 ubiquitination appears to be controlled by the ubiquitin-specific protease-9,^56^ and they are both associated with Alzheimer’s disease.^22,23,51^

We found that *Mark4* ablation caused a dramatic reduction in AT8-positive and thioflavin S-positive tau (Figs 4 and 5). Although the AT8 sites, Ser202 and Thr205, are not direct MARK4 phosphorylation targets,^57^ MARK4 may affect their phosphorylation via other kinases. GSK3β can phosphorylate more than 15 tau phosphorylation sites, including AT8 sites,^8,58-61^ and is believed to be essential for tau toxicity *in vivo*.^62^ AT8 sites are also targets of Cdk5,^8,63^ and MARK4 enhances tau phosphorylation mediated by Cdk5.^27^ In *Drosophila*, it was shown that tau phosphorylation at Ser262 and Ser356 by Par-1, the fly homolog of MARK, primed tau hyperphosphorylation at GSK3β sites.^11^ Primed tau phosphorylation was further confirmed in mammalian primary neurons.^64^ We observed a 2-fold AT8 signal reduction in PS19 mice upon *Mark4* ablation (Fig. 4C), which may, in part, be due to the effects of a lower level of primed phosphorylation caused by decreased tau phosphorylation at Ser356. Tau phosphorylation at Ser356 affects its interactions with molecular chaperones that enhance tau aggregation,^65^ suggesting that altered interactions with molecular chaperones may contribute to decreasing the insoluble levels of tau in a *Mark4* knockout background. Our results highlight a critical role of tau phosphorylation at Ser356 upstream of tau aggregation *in vivo*.

Finally, we found that *Mark4* knockout reduced astrogliosis to WT levels in the PS19 mouse model (Fig. 6), which may also contribute to ameliorating tau toxicity. Previous reports indicate that a reduction in microgliosis and astrogliosis attenuates brain atrophy without changing tau pathogenic phosphorylation,^47^ suggesting that gliosis affects the disease phenotype downstream of tau pathological perturbations. Interestingly, microgliosis was not significantly affected by *Mark4* knockout in PS19 mice (Fig. 6), and *Mark4* deletion reduced the number of active astroglia in the hippocampus of 9-month-old mice that did not express human P301S tau (Fig. 7). These findings point towards a physiological role of MARK4 in astroglia activation, independent of tau lesions. MARK4 has been reported to mediate the activation of NLPR3 inflammasomes, which constitute critical signalling platforms in bone marrow-derived macrophages of the innate immune system.^50^ It has been reported that loss of *Mark2* facilitates activation of microglia in culture and in the mouse brain.^66^ However, the functions in MARK2 in the astrocyte have not been reported. While all MARK family members are expressed in astrocytes (Human Protein Atlas, proteinatlas.org^67^), we observed that MARK4 ablation decreased astroglia activation (Figs 6 and 7), suggesting functions of MARK4 in astrocytes are not redundant to other members. *Mark4* ablation may ameliorate tau-induced neurodegeneration by not only reducing pathological tau modifications but also suppressing astrogliosis. Further investigation of glial changes in response to MARK4 suppression in other neurodegenerative disease models may reveal novel aspects of the mechanisms underlying astroglia activation in disease pathogenesis.

In this study, we demonstrated the critical role of MARK4 in tau-induced neuropathology and indicated that reducing MARK4 activity is sufficient to ameliorate tau pathology. In particular, *Mark4* knockout in PS19 mice prolonged survival and restored memory to WT levels, which was accompanied by reduced synapses and dendritic loss, disease-associated tau phosphorylation, tau aggregation and astrogliosis. Our results suggest that MARK4 is a reasonable target for the identification of novel tauopathy treatments.

## Supplementary Material

fcae136_Supplementary_Data

## Data Availability

The data sets used and/or analysed in this study are available from the corresponding author upon request.
