# Supplementary material for: Mark4 ablation attenuates pathological phenotypes in a mouse model of tauopathy
Source: Brain Commun. 2024 Apr 17;6(3):fcae136. doi: 10.1093/braincomms/fcae136 (PMC11073748; doi:10.1093/braincomms/fcae136)
Supplement: fcae136_Supplementary_Data [file fcae136_supplementary_data.pdf]

Supplementary Table 1. Number of mice in F2 generation

Genotype	Total	Ataxia	Survived, %
Wild type	13	0	100
Mark4$^{+/-}$	13	0	100
Mark4$^{-/-}$	17	0	100
PS19	16	5	69
PS19:*Mark4*$^{+/-}$	30	4	87
PS19:*Mark4*$^{-/-}$	14	2	86

Supplementary Table 1. The number of mice bred for experiments was obtained by crossing PS19:*Mark4*$^{+/-}$ and *Mark4*$^{+/-}$ mice. All mice were males.

Figure S1

Supplementary to figure 1

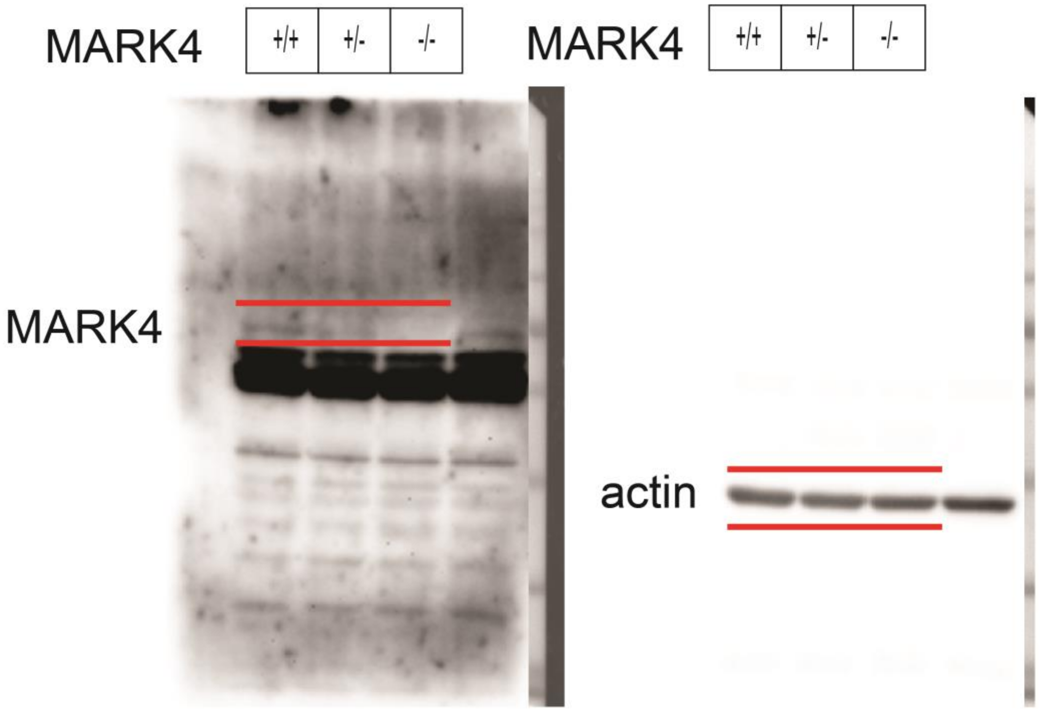

Supplementary Figure 1. Full- size and uncropped blots for Figure 1b.

Figure S2

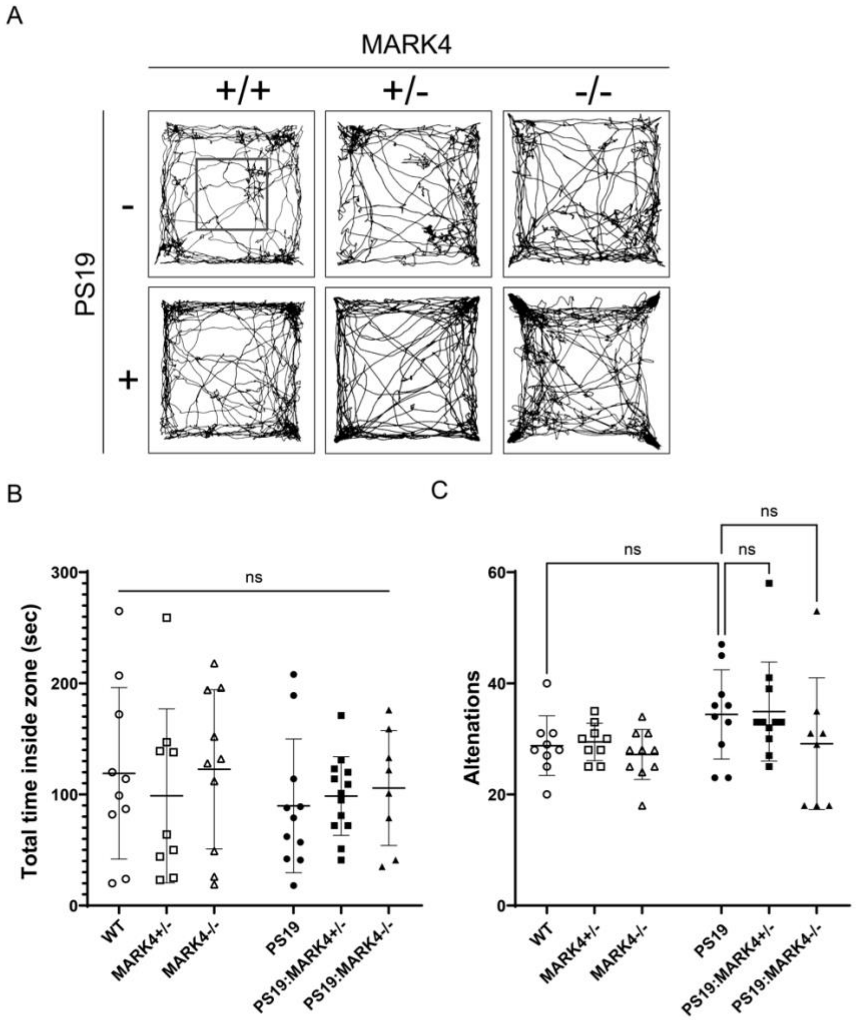

Supplementary Figure 2. *Mark4* knockout did not affect the activity of wild-type and PS19 mice. (A) Trajectories of 9-month-old mice, running for 10 min in the open field test. **(B)** Time spent in the 20×20 cm middle zone (red square) of the arena for over 10 min during the open field test. Data represent the mean $\pm$ SD. $N = 8$ to $N = 13$ mice/group. Two-way ANOVA with Tukey's multiple comparisons test. **(C)** Numbers of total alterations in the Y-maze spontaneous alteration test. Data represent the mean $\pm$ SD. $N = 8$ to $N = 11$ mice/group. ns, not significant; two-way ANOVA with Tukey's multiple comparisons test.

Figure S3

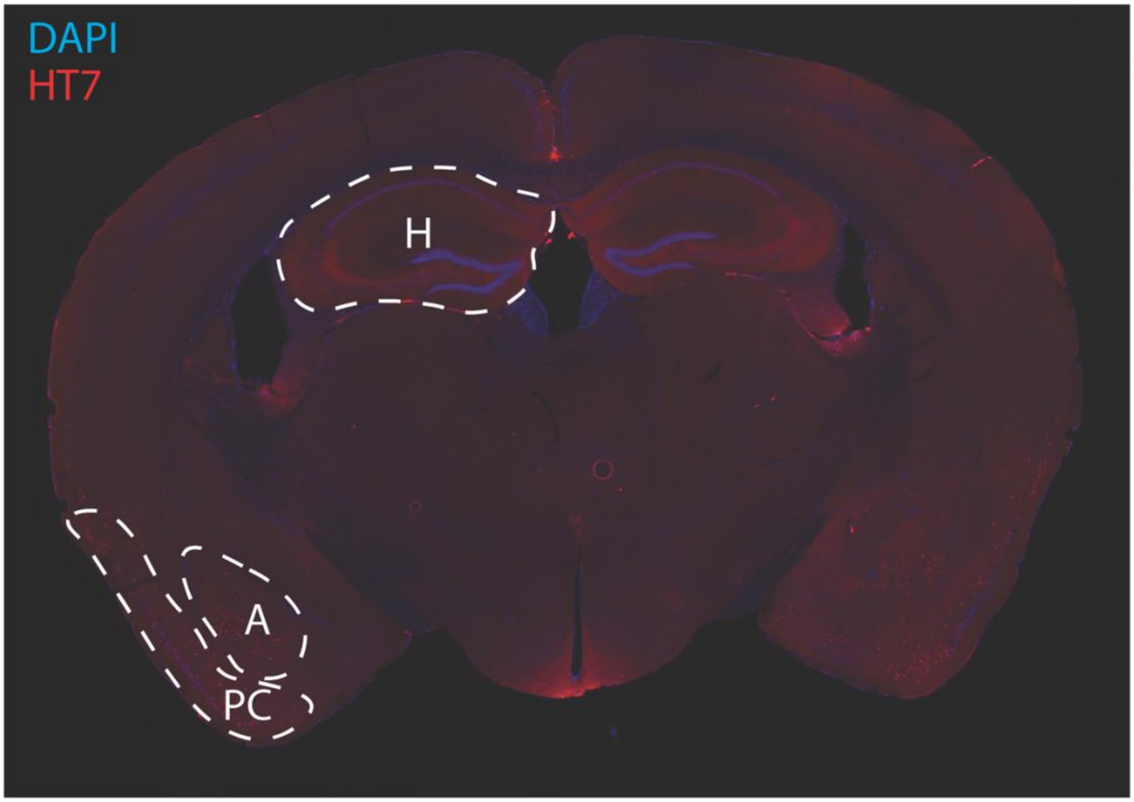

Supplementary Figure 3. The nine-month-old PS19 mouse brain, coronal section representative image. Blue – DAPI, red – HT7 antibody. Highlighted regions by dashed lines: H – hippocampus, A – amygdala, and PC – piriform cortex.

Figure S4

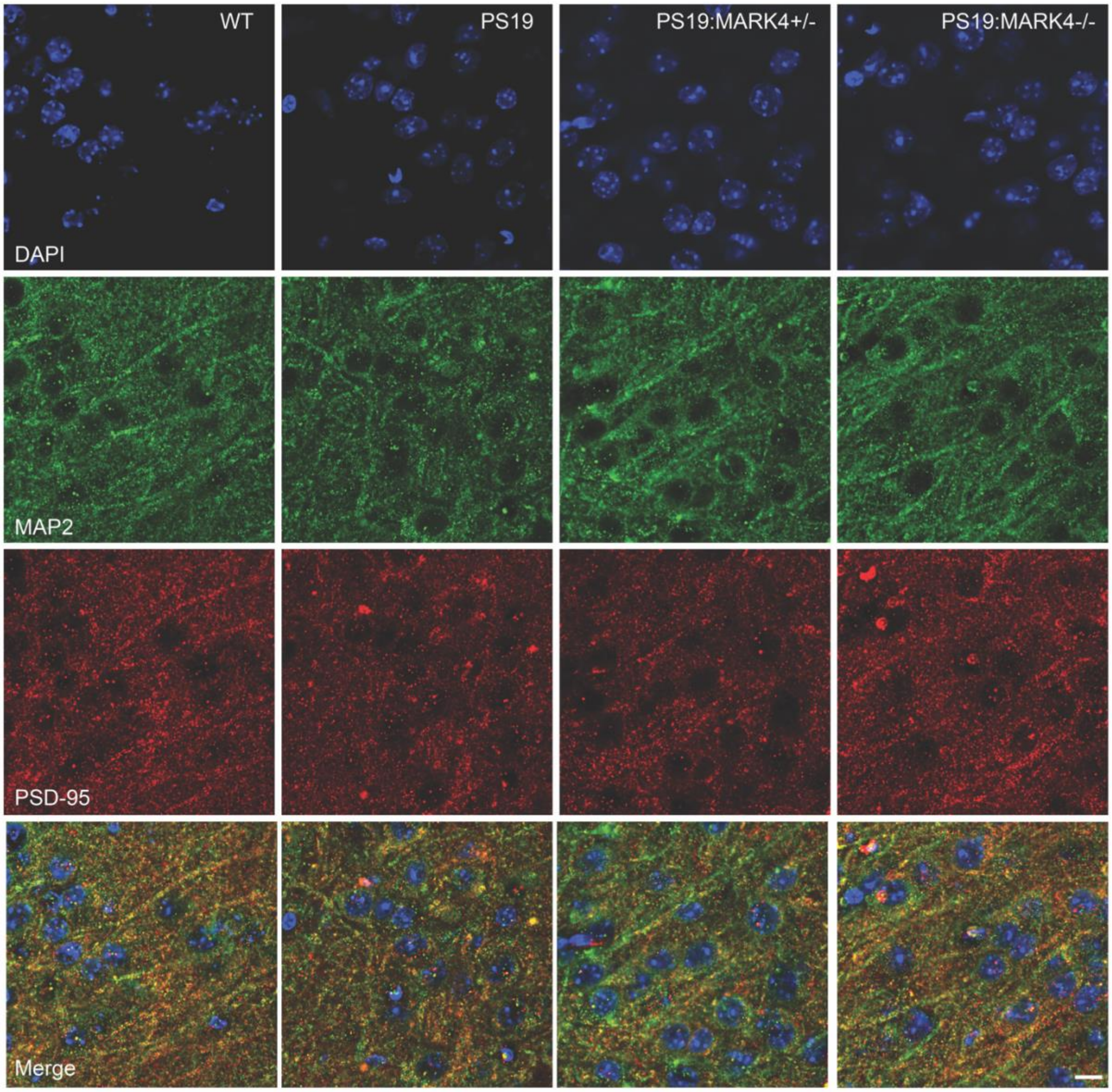

Supplementary Figure 4. Representative confocal microscopy images of neurons in the piriform cortex region stained with PSD-95 (red) and MAP2 (green) antibodies and DAPI (blue). Scale bar, 10 μm.

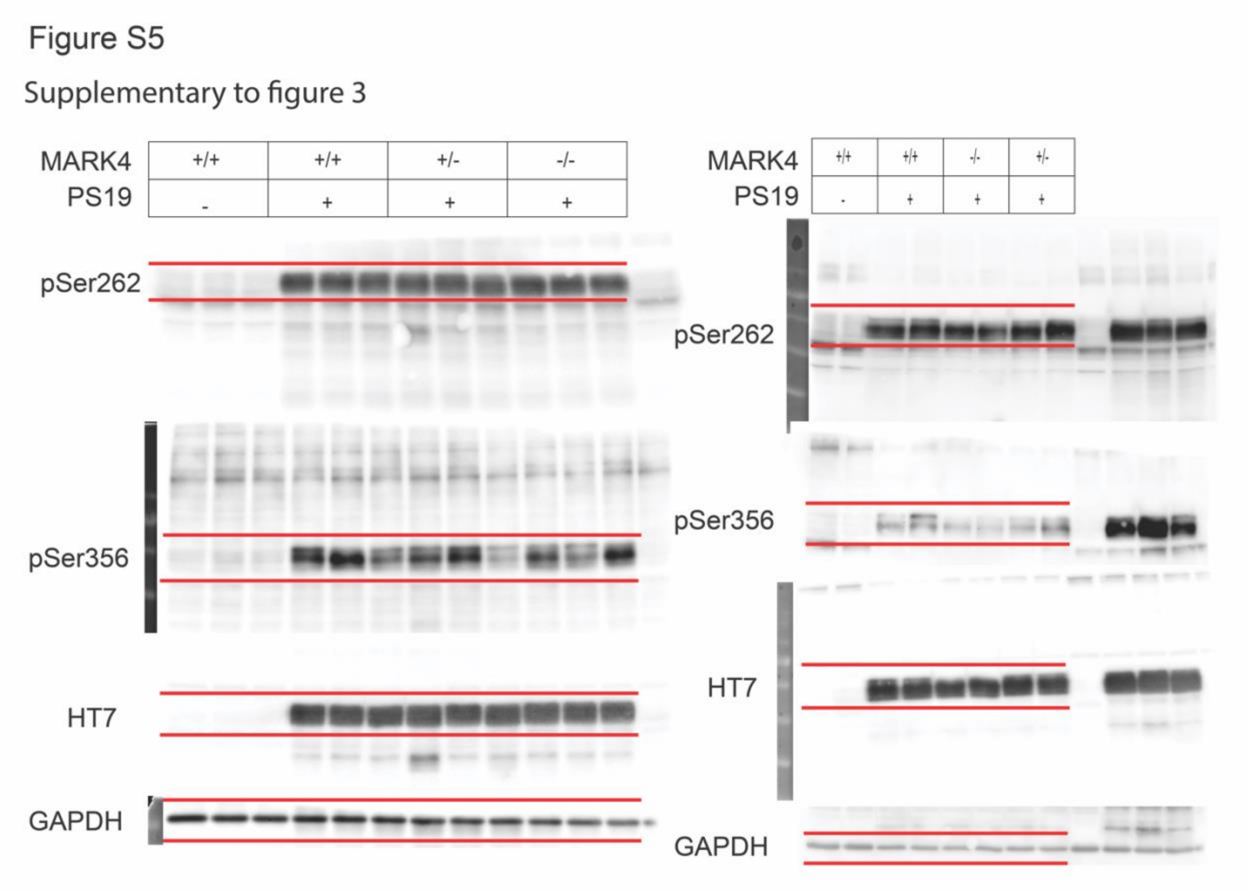

Supplementary Figure 5. Full- size and uncropped blots for Figure 3. Bands between red lines were analyzed.

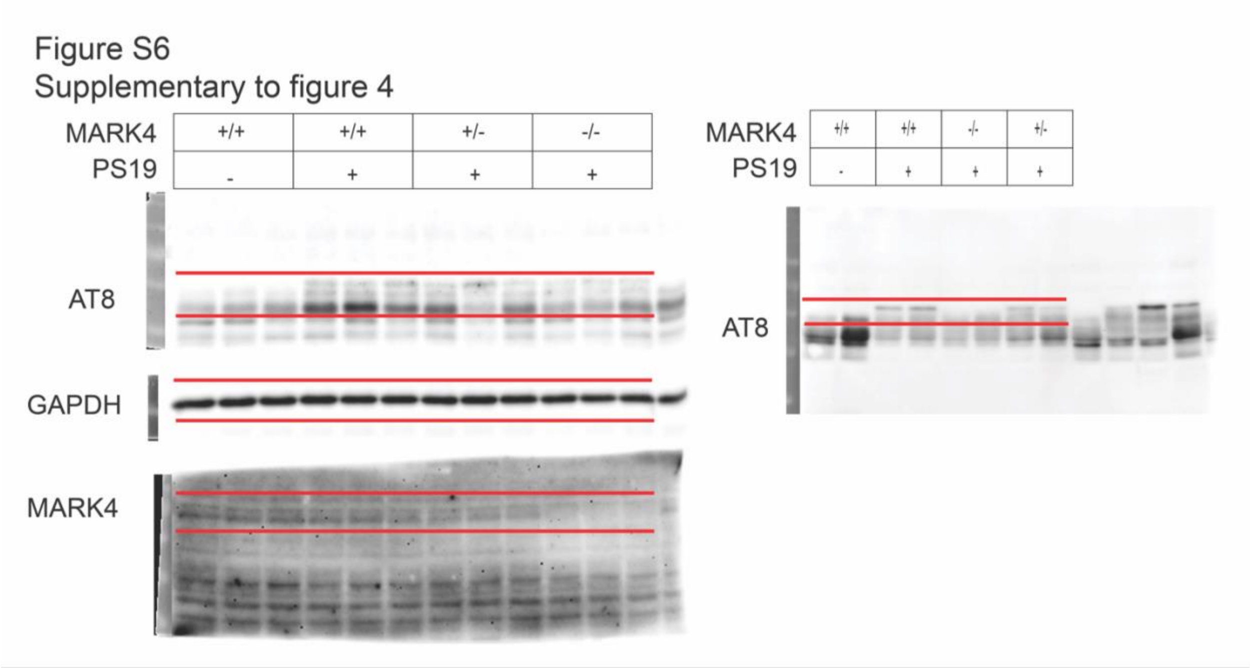

Supplementary Figure 6. Full- size and uncropped blots for Figure 4. Bands between red lines were analyzed.

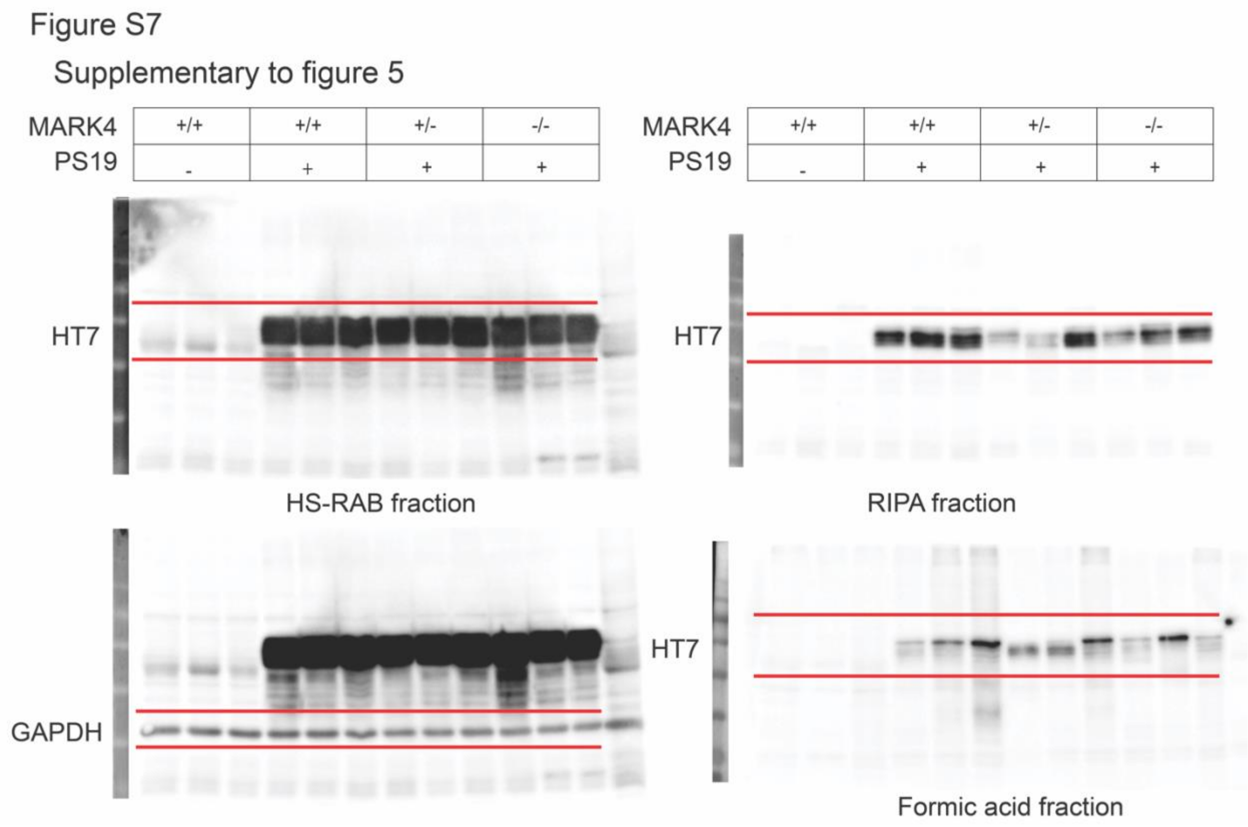

Supplementary Figure 7. Full- size and uncropped blots for Figure 5. Bands between red lines were analyzed.

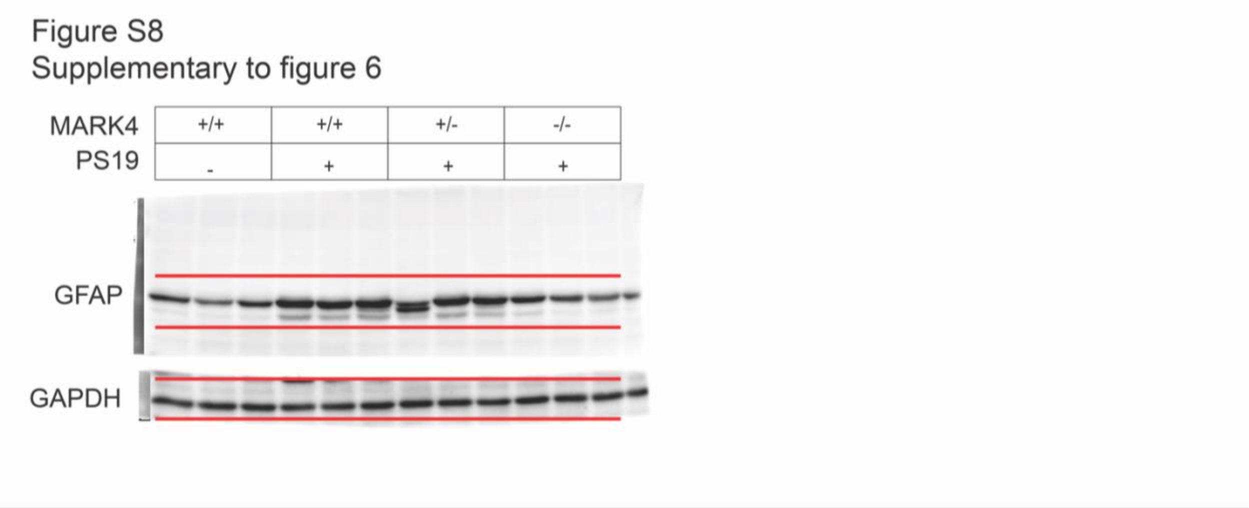

Supplementary Figure 8. Full- size and uncropped blots for Figure 6. Bands between red lines were analyzed.
